# Models to Tailor Brain Stimulation Therapies in Stroke

**DOI:** 10.1155/2016/4071620

**Published:** 2016-02-23

**Authors:** E. B. Plow, V. Sankarasubramanian, D. A. Cunningham, K. Potter-Baker, N. Varnerin, L. G. Cohen, A. Sterr, A. B. Conforto, A. G. Machado

**Affiliations:** ^1^Department of Biomedical Engineering, Lerner Research Institute, Cleveland Clinic Foundation, Cleveland, OH 44195, USA; ^2^School of Biomedical Sciences, Kent State University, Kent, OH 44242, USA; ^3^Human Cortical Physiology and Stroke Neurorehabilitation Section, National Institute of Neurological Disorders and Stroke, NIH, Bethesda, MD 20892, USA; ^4^University of Surrey, Guildford, Surrey GU2 7XH, UK; ^5^Neurology Clinical Division, Neurology Department, Hospital das Clinicas, Sao Paulo University, 05508-090 Sao Paulo, SP, Brazil; ^6^Hospital Israelita Albert Einstein, 05652-900 Sao Paulo, SP, Brazil; ^7^Center for Neurological Restoration, Neurosurgery, Neurological Institute, Cleveland Clinic Foundation, Cleveland Clinic, Cleveland, OH 44195, USA

## Abstract

A great challenge facing stroke rehabilitation is the lack of information on how to derive targeted therapies. As such, techniques once considered promising, such as brain stimulation, have demonstrated mixed efficacy across heterogeneous samples in clinical studies. Here, we explain reasons, citing its one-type-suits-all approach as the primary cause of variable efficacy. We present evidence supporting the role of alternate substrates, which can be targeted instead in patients with greater damage and deficit. Building on this groundwork, this review will also discuss different frameworks on how to tailor brain stimulation therapies. To the best of our knowledge, our report is the first instance that enumerates and compares across theoretical models from upper limb recovery and conditions like aphasia and depression. Here, we explain how different models capture heterogeneity across patients and how they can be used to predict which patients would best respond to what treatments to develop targeted, individualized brain stimulation therapies. Our intent is to weigh pros and cons of testing each type of model so brain stimulation is successfully tailored to maximize upper limb recovery in stroke.

## 1. Introduction

Stroke is a leading cause of long-term adult disability [1]. Of its 7 million survivors in the United States, a majority require help with self-care and report restriction in daily activities [2, 3]. Chronic paresis of the hemiplegic upper limb is at the core of stroke-related disability because >78% patients never reach age-based norms, and 67% perceive upper limb disuse disabling despite rehabilitation [4, 5]. Several adjunctive therapies have been proposed to maximize and accelerate rehabilitative outcomes of the upper limb. One of the most popular techniques involves stimulating the motor cortices. Stimulation can be applied using invasive, implanted electrodes [6] or noninvasive techniques that deliver currents via electromagnetic induction (transcranial magnetic stimulation, TMS) or direct current application over the scalp and skull (transcranial direct current stimulation, tDCS) [7, 8]. The essential premise is that electrically stimulating the motor cortices could serve to potentiate plasticity that underlies recovery of the paretic upper limb [7, 9–19]. Several studies have demonstrated promise of brain stimulation towards affecting recovery. Therapeutic effect sizes range anywhere from 10% to even 30% relative to baseline [7].

Despite the promise, neither invasive nor noninvasive stimulation is used in outpatient clinical settings. The approach has shown mixed efficacy in recent clinical studies [6, 15, 17, 20, 21]. A key limitation, as is believed, is the use of generic, unvarying methodology; given the heterogeneity that is characteristic of stroke, several groups acknowledge that a one-type-suits-all methodology would naturally be variable [15, 16, 22]. Ward et al. best summarize a potential solution [23]: “Stroke patients are a heterogeneous group. By explaining this heterogeneity between stroke patients in terms of measurable parameters, it should be possible to predict the response to treatments with known mechanisms and therefore to target individuals appropriately.” It is in this context that the present report seeks to propose potential frameworks and models that could help stratify patients for tailored or personalized cortical stimulation therapies. In the absence of prospective data, hypotheses here are still conceptual, hence by no means complete to represent possible means to personalize stimulation.

The present report arrives at a discussion of the potential frameworks to tailor stimulation by discussing the following pieces of evidence:

1. What is the existing approach to cortical stimulation in stroke?
2. When, and why, does the existing approach fail?
3. What are the likely alternate approaches to support recovery?
4. How does one determine the alternate substrate that would most likely benefit an individual's recovery?

## 2. What Is the Existing Approach to Stimulation in Stroke?

The current approach believes that plasticity of the primary motor cortex (M1) in the ipsilesional (affected) hemisphere most impacts recovery and that intact, contralesional cortices (in the unaffected hemisphere) compete with and inhibit ipsilesional plasticity [13, 24–29]. Therefore, the approach calls for facilitating excitability of ipsilesional M1 and suppressing excitability of contralesional M1. The premise that plasticity of residual M1 supports recovery and intact contralesional cortices inhibit ipsilesional plasticity emerges from two critical sources of evidence.

### 2.1. Evidence That Ipsilesional M1 Is Central to Plasticity for Stroke Recovery

M1 is considered the most critical part of the executive motor system adapted especially for selectively activating muscles involved in skilled upper limb motor behavior [30, 31]. Tremendous neurophysiologic and neuroimaging evidence has helped formulate the premise. Two landmark investigations have presented some of the earliest accounts of how plasticity of M1 underlies recovery in stroke. Nudo and colleagues demonstrated in nonhuman primate models of stroke that over the course of spontaneous recovery and learning-based skill training territories within ipsilesional M1 remap [32, 33]. Territories devoted to different parts of the upper limb were mapped at first using intracortical microstimulation (ICMS). After an infarct destroyed a substantial portion of the territories devoted to the distal forelimb, Nudo and colleagues witnessed functional remapping in M1. Residual representations devoted to the distal forelimb diminished while their territories came to be occupied by representations of the more proximal and less-affected elbow/shoulder segments during the course of spontaneous recovery [32]. When animals were trained on skilled tasks involving the affected distal forelimb, however, M1 remapped differently. This time residual distal forelimb representations expanded into territories occupied previously by the proximal forelimb [32–34]. Such rapid shifts in peri- and ipsilesional territories of M1 that have the potential to reverse sequela of disease have become the most popular substrate to target with cortical stimulation.

Evidence from functional neuroimaging reinforced these early theories derived from neurophysiology. Serial functional MRI (fMRI) or Positron Emission Tomography (PET) revealed how activation patterns evolve in humans from hyperacute to chronic poststroke recovery, speaking to the importance of ipsilesional M1 [35–37]. fMRI and PET capture real-time activity of the brain during movement of the paretic limb. As individuals recover hand function, activation becomes localized to ipsilesional sensorimotor cortex and ipsilesional M1; individuals with incomplete recovery, however, continue to demonstrate bilateral and contralesional activation ipsilateral to the moving paretic limb [35]. From these studies a consensus emerged that boosting plasticity within ipsilesional M1 could profoundly impact recovery.

Besides cortical activation, evidence pertaining to physiologic excitability and output of pathways too validated the role of ipsilesional M1 in recovery of the paretic upper limb. One can typically capture excitability and output of pathways from M1 using transcranial magnetic stimulation (TMS) [38–40], akin to ICMS in animal models. TMS is applied to a single site or to a grid of several sites. With rehabilitation, typically, excitability and output would improve thresholds to activate residual pathways that would decrease; map/grid sites devoted to paretic muscles would increase, and excitability of interneuronal circuits within ipsilesional M1 would increase [38–43]. Therefore, the current standard of cortical stimulation emphasizes boosting excitability of ipsilesional M1 and its pathways to the paretic limb to boost benefit from rehabilitative therapies.

### 2.2. Evidence That Contralesional Cortices Oppose Recovery

While evidence favoring the significance of ipsilesional M1 was becoming prominent, evidence for the negative influence of contralesional cortices was also emerging. Classical studies of functional imaging demonstrated that activation of contralesional motor cortices accompanied movement of the paretic limb in patients with incomplete recovery [35, 37, 44–46]. Landmark neurophysiologic studies validated the claims. In a study that employed bihemispheric TMS, where TMS to contralesional M1 was applied a few milliseconds prior to TMS to ipsilesional M1, Murase et al. explained how contralesional M1 inhibited output from the ipsilesional M1 via transcallosal effects [24]. Conditioning pulses to contralesional M1 suppressed activity evoked in paretic muscles with TMS to ipsilesional M1. Greater suppression was associated with poorer recovery of the paretic limb. While it should be remembered that Murase et al. studied patients who were recovered enough to perform distal finger motor task, their study of interhemispheric inhibition set one of the strongest bases for present-day brain stimulation therapy. Evidence that in a group of relatively well-recovered patients the contralesional M1 levies strong, persistent inhibition upon ipsilesional M1 via callosal interactions has shaped our current philosophy of stroke motor recovery and underlying neurophysiology. As such, the current standard of cortical stimulation emphasizes inhibiting excitability of contralesional M1 to disinhibit and boost output of weak, ipsilesional M1.

Taken together, several lines of evidence from animal and human studies, based on neurophysiology and neuroimaging, have informed the basis of current standard for cortical stimulation therapy. The current standard is based on the model of interhemispheric inhibition, the idea that ipsilesional M1 is central to most impactful plasticity while its homologue opposes recovery via transcallosal interactions in relatively well-recovered patients. Therefore, the current standard seeks to restore interhemispheric balance to maximize recovery by boosting excitability of ipsilesional M1 and inhibiting excitability of contralesional M1.

## 3. When and Why Does the Existing Approach Fail?

If ipsilesional M1 is a critical resource for plasticity and contralesional M1 opposes recovery, then why does the current standard of stimulation fail to benefit many? The answer we believe lies in the nature of stroke-related injury and consequent effects on physiology that deviate from classical tenets of the model of interhemispheric inhibition.

### 3.1. The Nature of Stroke-Related Injury

Ipsilesional M1 or its corticospinal pathways are damaged in ~96% of patients experiencing a typical middle cerebral artery stroke [15, 16, 47–50]. In fact, damage is so extensive in 58–83% of patients that stimulating ipsilesional M1 fails to evoke a response in muscles of the paretic upper limb [6, 16, 50]. It is thus not surprising that patients with extensive damage and deficit respond poorly to stimulation of ipsilesional M1, whereas outcomes are fairly homogenous and promising in those with minimal damage and impairment [23, 38, 46, 51–54]. This discrepancy may also explain why stimulating ipsilesional M1 is found to be frequently effective in smaller pilot studies [25, 55] than in large-scale pivotal trials [6, 17]. For instance, invasive stimulation of ipsilesional M1 was witnessed to be advantageous for rehabilitative outcomes in phase I/II studies [48, 49, 55], but benefits failed to translate into later phase III pivotal trial [6]. It was reasoned that a majority of patients in the early trials had preserved pathways where stimulation of ipsilesional M1 could evoke movements in the paretic limb, but the phase III study enrolled a majority without evidence of such sparing [16, 50]. Along similar lines, when damage to the territory of ipsilesional M1 is considered in different studies, benefits become weak and variable with cortical lesions affecting the ipsilesional territory [56, 57]. As such, since large-scale studies include larger number of patients with heterogeneous damage and disability, variability in lesions and degree of injury to ipsilesional M1 and pathways weakens the effectiveness standard stimulation. Since the presumed substrate for plasticity, and the target for current stimulation therapies, ipsilesional M1, is affected most commonly in typical injuries, a singular approach to stimulation would inevitably be variable in affecting recovery [16, 22].

### 3.2. Challenges to the Classical Model of Interhemispheric Inhibition

The current standard also varies because its underlying model, the model of interhemispheric inhibition, deviates under many circumstances. For example, recovery in subacute, subcortical stroke is associated with gains in ipsilesional excitability, but interhemispheric inhibition remains stable and symmetric. Stinear et al. studied patients with subcortical stroke who had experienced no damage to the cortical territory of ipsilesional M1; in such cases, the most notable contribution to recovery came from gains in ipsilesional excitability [58], while interhemispheric balance was not disrupted, nor did it evolve with recovery. The model also fails to explain why inhibiting excitability of affected motor cortices reduces hypertonicity and improves function of the paretic upper limb, when according to the model facilitating excitability would be expected to have such an effect [59]. The model also does not explain why inhibiting excitability of contralesional M1 reinstates deficits of the paretic upper limb in patients with greater impairment [60]. It becomes conceivable that contrary to the model's premise, which originated in relatively well-recovered patients, interhemispheric inhibition from contralesional motor cortices is not significant in patients with greater impairment of the paretic limb. The model also appears to deviate based on the extent and nature of injury and behavioral influences. For instance, learned “nonuse” of the paretic limb and injury to cortical *γ*-amino butyric acid (GABA) interneurons that interact with callosal neurons affects interhemispheric inhibition and alleviation of inhibition with recovery. After stroke, patients typically learn to rely on use of their nonparetic limb in order to compensate for failures they experience with the use of the paretic limb, which exaggerates “learned nonuse” of the paretic limb and hampers recovery. Blicher et al. offered rehabilitative therapy, where they required patients to focus on using their paretic upper limb during restraint of the nonparetic limb [61]. They found that GABA levels and interhemispheric inhibition decreased after therapy, in association with functional improvement, but changes were individual-specific. Patients with greatest nonuse and those with high interhemispheric inhibition experienced greatest decreases in GABA and tremendous gains in therapy. Patients with damage to GABA neurons in the ipsilesional cortices did not show gains with therapy.

Therefore, the current standard of stimulation fails to benefit many patients likely because ipsilesional M1 and its pathways are commonly injured, which affects the ability to facilitate ipsilesional excitability. Additionally, the model of interhemispheric inhibition deviates in the presence of subcortical injuries, learned nonuse of the paretic limb, and loss of GABAergic interneurons, which weakens the basis of standard stimulation therapy.

## 4. What Are the Likely Alternate Sources That Could Support Recovery?

Even though M1 is considered critical to the executive motor system [30, 31], scope for its plasticity is remarkable only in patients without significant injury, while, in patients with damaged M1 or pathways, alternate sources can express plasticity to contribute to recovery. Since motor cortical areas can act in parallel to generate and control distal limb movements [30], it becomes conceivable that they have the ability to substitute for each other in cases of injury. As such, when standard stimulation fails, alternate areas may serve as new sources of recovery. These areas include the following.

### 4.1. Ipsilesional Premotor Areas

Alternate substrates for plasticity typically include higher-order ipsilesional regions, like premotor and supplementary motor cortices (PMC and SMA) [51], known collectively as premotor areas. Their plasticity can be meaningful [62–64] because they constitute more than 60% of the frontal cortex projecting to the spinal cord [65]. Although originally it was believed that they do not contribute to corticospinal pathways [26], Dum and Strick [65] showed these areas contribute ~40% of pathways to the hand, independently of M1. And even though only a small number of premotor pathways are actually connected to spinal interneurons for finger muscles and their cortical cells have smaller muscle field size, their contribution still matches or exceeds contribution from M1 [65, 66] and can undergo structural plasticity like better myelination [67, 68]. As such, ipsilesional premotor areas form direct, parallel modules for control of distal forelimb independent of M1.

**Figure 1:**
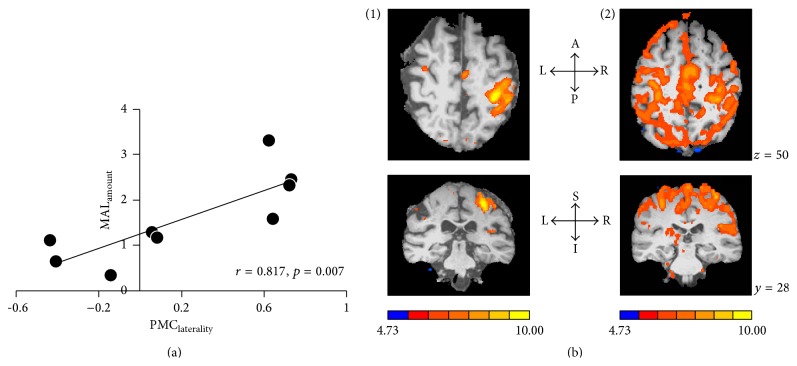
Role of ipsilesional premotor areas: from our work in Cunningham et al. [38]. PMC: premotor cortex (synonymous with PMd here); MAL: motor activity log. The figure explains the potential of ipsilesional higher motor areas including ipsilesional PMd in recovery. Patients with wide-ranging baseline impairments undergo functional MRI during movement of the paretic hand. Activation of ipsilesional versus contralesional cortices is computed using a laterality index, where a higher positive value suggests cortices contralateral to the paretic limb are activated. (a) demonstrates that patients who show better laterality for PMd, that is, greater activation of ipsilesional versus contralesional PMd (*x*-axis), perceive less disability in using their paretic hand (*y*-axis). Perception of disability is signified using a popular scale, known as MAL. Values on MAL range anywhere from 0 to 5, where 5 signifies no disability in the use of the paretic hand. (b) presents an illustration of subjective examples. Two patients, labeled 1 and 2, underwent fMRI during movement of their paretic hand. Images show fMRI activation in transverse (top) and coronal (bottom) planes. For simplicity, the lesioned hemisphere is shown to the right of each image. Patient 1 demonstrates focused activation of ipsilesional PMd that coincides with greater laterality, while patient 2 shows weaker laterality because activation of most regions is bilateral. MAL scores for patients 1 and 2, respectively, are 2.3 and 0.66. Therefore, patient 1 who perceived lesser disability in using the paretic hand showed greater activation of ipsilesional PMd, though patient 2 with extreme perception of disability activated multiple other regions. Patients who recover, albeit incompletely, can rely on plasticity of their ipsilesional premotor areas.

Not only do they offer alternate motor output, but their cortical territories can remap to assume functions typically served by damaged M1. For instance, when majority of distal forelimb representation in M1 is destroyed, premotor areas can show remapping of the corresponding representation by up to ~50% [69, 70]. Similarly, with damage to M1 and its corticospinal pathways, patients can exhibit task-related fMRI activation within ipsilesional premotor areas that increases proportionally with the degree of the injury [46, 62, 71–73]. We have found as well that activation increases linearly with better perception of disability of the paretic limb [46] (Figure 1). Premotor cortices can also pair with associative posterior parietal cortices as in the case of learning to control a brain-computer interface with a completely paralyzed extremity [74]. With long-term learning, intensity of activating PMC but not ipsilesional M1 changes suggesting premotor networks improve in efficiency over the course of recovery [46, 75]. Such remapping is causal, not just epiphenomenal; inactivating premotor areas but not the ipsilesional M1 [76] reinstates motor deficits in recovered animals and humans [76–79].

The ability of ipsilesional premotor areas to remap can be ascribed to their flexible organization and connectivity. For instance, we find SMA possesses integrated representations just like M1, but PMC presents differentiation in distal and proximal representations like sensory cortices [82]. Ipsilesional premotor areas also share strong connectivity with ipsilesional M1. Wu et al. recently employed dense-array EEG to study coherence of activity between these regions, finding that ipsilesional premotor-M1 connectivity was the strongest predictor of chronic motor status, and the change in their connectivity evolved with gains in therapy [83]. Therefore, though, in most cases of mild damage, recovery can rely on ipsilesional M1 and its residual pathways, with greater damage to large-diameter fibers from M1, small diameter fibers from PMC and SMA that are more resistant to ischemia can offer independent parallel nonprimary motor loops [84]. This is not to say that recruitment of ipsilesional premotor areas can help achieve complete recovery (Figure 1). Yet, these instances suggest that clinical improvement can occur in patients with (near) complete damage to M1 and its corticospinal pathways via “reorganized” albeit limited pathways from ipsilesional premotor territories.

An important caveat needs to be considered, however. The potential of ipsilesional premotor areas is evident more consistently in animal models with homogenous focal infarcts [77, 85, 86] or in humans with focal injuries that spare PMC and SMA [62, 79, 87] and/or posterior portions of the posterior limb of the internal capsule where their corticospinal tracts converge. But, in a typical middle cerebral artery stroke, where 96% of patients experience white matter damage at the level of periventricular and internal capsular regions [47], it is less likely that a lesion affecting pathways from ipsilesional M1 would spare pathways from PMC and SMA. Injury to tracts from ipsilesional M1 and ipsilesional PMC or SMA is not remarkably different [88, 89]. With damage limited to pathways from ipsilesional M1, one can anticipate other ipsilesional areas could become meaningful for recovery, but given that lesions are heterogeneous, the potential for plasticity offered by alternate ipsilesional substrates would theoretically remain uncertain and inconsistent. As such, in one of our most recent clinical studies, patients receiving stimulation to facilitate ipsilesional premotor areas during rehabilitation recovered more than patients receiving rehabilitation alone, but benefits were variable to a certain degree [90].

### 4.2. Contralesional Motor and Higher Motor Areas

Liu and Rouiller have proposed a gradient of plasticity, which varies with the extent of stroke-related injury. When damage is small and pathways are partially spared, it is possible for perilesional M1 and ipsilesional premotor areas, like PMC and SMA, to reorganize in a way that supports recovery. But, with larger lesions affecting most of frontal cortices and pathways, there is little option but to rely on plasticity of intact, contralesional cortices [76, 91, 92]. For instance, in a randomized clinical study involving patients with little function (Upper Extremity Fugl-Meyer = 9–12), improvements with 12 weeks of training were associated with activation in contralesional premotor areas rather than ipsilesional M1 [63]. Such contralesional plasticity has a causal influence and is not simply a characteristic of patients with greater disability. For example, when excitability of contralesional hemisphere is suppressed with traditional brain stimulation, deficits become reinstated in patients with greater disabilities. This too serves as a deviation from the classical notion of interhemispheric inhibition suggesting contralesional motor cortices are adaptive for recovery at least in patients with greater damage and disability [60, 93–96].

Of all the contralesional cortices, contralesional dorsal premotor cortex (PMd) has greatest likelihood to support recovery because of the following.

(a) With greater impairment, cPMd can exert more causal influence upon recovery than other contralesional motor cortices. During performance of a reaction time task at the paretic upper limb, Johansen-Berg et al. and Bestmann et al. separately suppressed cPMd, cM1, and other cortices using TMS. Suppression of cPMd significantly slowed movement of the paretic limb. Slowness was more prominent in patients with greater impairment of the paretic limb [94, 95]. Therefore, Johansen-Berg et al. and Bestmann et al. concluded that cPMd exerts a more causal influence than other cortices in the recovery of patients with greater impairment of the paretic limb.

(b) cPMd can exert a causal influence by limiting inhibition it imposes upon the paretic limb. Bestmann et al. sought to understand what constituted a causal influence from cPMd. They conducted two sets of experiments. In one set of experiments, they tested neurophysiologic inhibition imposed from cPMd upon ipsilesional M1 using bihemispheric TMS. In the second experiment, they applied TMS to cPMd during grip tasks involving the paretic limb as they acquired fMRI. Using bihemispheric TMS, Bestmann et al. found that interactions between cPMd and ipsilesional M1 were predominantly inhibitory in patients with minimal impairment, which aligned with the traditional model of interhemispheric inhibition. But, in patients with greater impairment, TMS to cPMd led to less inhibition and even facilitation of output from ipsilesional M1. When Bestmann et al. repeated TMS during fMRI, they found that TMS to cPMd facilitated activation with ipsilesional M1 in patients with greater impairment of the paretic limb. Therefore, cPMd could exert a causal influence upon ipsilesional M1 via physiologic interhemispheric interactions; cPMd could lessen its inhibition on ipsilesional M1 to support recovery especially in patients with greater impairment of the paretic limb.

cPMd could likely modulate its inhibition upon ipsilesional M1 because it shares strong callossal connectivity with homologous as well as heterologous cortices. Unlike M1 that shares some of the weakest callossal connections, PMd is densely connected with opposite PMd and opposite M1 [97, 98]. PMd shares extensive callossal connectivity potentially since it is involved in mediating abstract higher-order movement planning for bilateral movements [98, 99].

**Figure 2:**
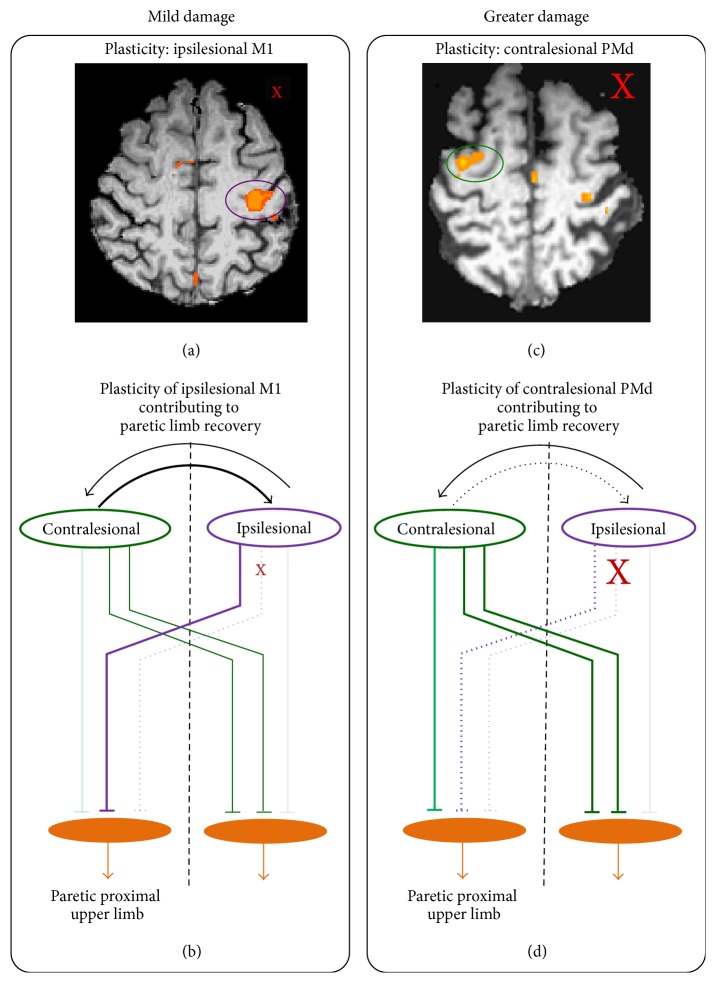
How contralesional PMD contributes to recovery of the paretic upper limb. Plasticity of ipsilesional M1 (iM1) is best evident in patients who are mildly impaired and have little damage to iM1 and corticospinal pathways because (a) they can feasibly recruit ipsilesional M1 in movement of the paretic upper limb in functional MRI (fMRI) and (b) can increase output of spared ipsilesional pathways (bold purple lines) to support the paretic limb. (c) Since, with greater damage, plasticity of ipsilesional M1 or any ipsilesional substrates is less likely, these patients recruit contralesional PMd in movement of the paretic limb. (d) Contralesional PMd reduces its inhibition on weak ipsilesional M1 (dotted black lines) so it partially supports paretic limb recovery (bolder, dotted purple lines). Also, contralesional PMd offers ipsilateral pathways (green) (uncrossed corticospinal and brainstem-mediated reticulospinal) to the proximal paretic limb to help recover [38, 80].

(c) cPMd can also have a causal role by offering ipsilateral pathways to the paretic limb in case of extreme damage to corticospinal pathways. With increasing damage to corticospinal pathways from ipsilesional M1, it is likely that contralesional motor cortices, including cPMd, can increase physiologic output of their* ipsilateral* pathways to the paretic limb [80, 81, 94, 100–108] (Figure 2). Ipsilateral pathways mainly support proximal and axial flexion [106, 109–111], so patients can at least recover functions like shrugging, elevation, and reaching, even if they cannot recover any distal control [106, 111–113]. cPMd gives more ipsilateral pathways than other contralesional cortices; these pathways are comprised of uncrossed corticospinal [114–117] and brainstem-mediated reticulospinal and rubrospinal connections [106, 111]. Therefore, with greater damage to corticospinal pathways from iM1, cPMd would be more likely to support recovery of the proximal paretic upper limb than other motor cortices.

Thus, in patients with substantial and variable damage and greater disability, contralesional areas especially the contralesional PMd could serve as more intact, consistent sources for plasticity to support recovery.

### 4.3. Other Substrates

Although the focus in clinical studies has been on stimulation of cortices, alternate substrates may be meaningful to consider for future studies. For instance, the contralateral cerebellum may play a key role in poststroke recovery. Cortical insult such as stroke is associated with rapid decreases in metabolic activity of the contralateral cerebellum, a phenomenon that is called crossed cerebellar diaschisis [118, 119]. Patients with severe crossed cerebellar diaschisis present with worse outcomes [120, 121] likely due to lack of excitatory input to cortical perilesional areas. Reversing crossed cerebellar diaschisis, as our group has proposed, presents a unique opportunity for promoting stroke recovery. For example, we have demonstrated that potentiating activity of cerebello-thalamo-cortical pathways via chronic stimulation of the dentate (lateral cerebellar) nucleus can reverse crossed cerebellar diaschisis in an animal model of middle cerebral artery occlusion [122]. Compared to sham-treated animals, animals that receive five weeks of chronic stimulation demonstrate a modest but significant improvement in motor outcomes [123]. When stimulation is paired with forelimb training in a model of focal infarct localized to M1, recovery is more favorable [124]. Benefits are associated with perilesional plasticity [125] and significant remapping, where representations of affected forepaw reemerge in perilesional cortical territories. Markers of long-term potentiation are significantly expressed and number of perilesional synapses increases. While results to date indicate that chronic stimulation of the dentate nucleus may become a viable therapy to promote recovery after stroke, the therapy has not yet been tested in humans. Findings here represent an opportunity for cerebellar stimulation as an emerging therapy in stroke rehabilitation.

Cortical plasticity has largely been related to structural integrity and physiologic excitability of corticospinal tracts from ipsilesional M1 and premotor areas. However, integrity of the extrapyramidal descending tracts is important to consider as well [126]. The extrapyramidal descending tracts include the rubrospinal tract originating from the red nucleus. In primates, the rubrospinal tract has monosynaptic connections with motor neurons located in the cervical spinal cord [127–131] for control of both proximal and distal muscles of the forelimb [31, 132]. Following damage to corticospinal tracts, the red nucleus can undergo synaptic reorganization to offer alternate output to paretic forelimb via the rubrospinal tract [111]. Despite a prominent presence in the primate model, rubrospinal tract does not appear to have a key role in normal motor control in humans. In instances of stroke, however, where corticospinal tracts become substantially damaged, rubrospinal tracts can offer compensatory support. Using diffusion tensor imaging (DTI), Rüber et al. [133] reveal a shift in microstructural properties of bilateral red nuclei and rubrospinal tract in relation to motor function in patients with chronic stroke who otherwise have experienced damage to corticospinal tracts. In a more recent study, Zheng and Schlaug [134] demonstrate plastic changes in the rubrospinal tract but not in the corticospinal tract following 2 weeks of concurrent cortical stimulation and physical/occupational therapy for the paretic upper limb. Therefore, while corticospinal tracts are prime in predicting recovery, in patients with substantial damage, the otherwise latent rubrospinal tracts and parent red nucleus can express structural and physiologic plasticity to help mediate recovery of the paretic upper limb.

When discussing brainstem-mediated pathways, one cannot overlook the contribution of medial brainstem systems including the reticulospinal tracts. In primates, neurons of the reticular formation are primarily involved in reaching and gross upper limb movements. They can participate in movements of fingers even though only 30% as often as corticospinal tracts and with 20% the amplitude. But, in those with lesions to the corticospinal tracts, the reticulospinal neurons become the most important candidates for recovery. Recovered hand movements however are often incomplete and appear poorly fractionated [105, 135].

## 5. How to Determine the Alternate Substrate That Would Most Likely Benefit an Individual's Recovery?

Since the original promise of cortical stimulation therapies has become faint in light of contradictory findings [17, 20, 136, 137], it is now more important than ever to tailor the technique rather than offer it as a generic therapy. While several substrates other than ipsilesional M1 can express plasticity as explained above, the greatest challenge lies in determining which alternate substrate could maximize individual's potential for recovery. Here, we summarize several theoretical models that are proposed to explain how to personalize or tailor stimulation therapies.

### 5.1. Model Based on Individual's Response to Stimulation

*The essential premise* of such models is that stimulation should be individualized to targets that patients are most responsive to in systematic comparisons. Shah-Basak et al. [138] proposed and tested one such model in the treatment of aphasia. As in upper limb recovery, the theory of interhemispheric inhibition dominates the application of brain stimulation in aphasia. It is typically believed that left-hemispheric frontal-temporal activity should be enhanced while right-hemispheric activity should be suppressed [139]. However, influence of the right hemisphere is more complex and variable and cannot always be considered inhibitory [140, 141]. Given the complexity, Shah-Basak et al. [138] designed a systematic study to individualize stimulation. Patients received facilitation of left frontotemporal regions, inhibition of homologues on the right, facilitation of regions on the right, and inhibition of frontotemporal regions on the left in a repeated measures crossover study. Seven out of 12 patients responded to at least one form of stimulation. But, as anticipated, response varied. Three individuals responded to the traditional left-hemispheric facilitation, and 3 individuals responded to left-hemispheric inhibition, while one responded to right-sided inhibition.* Post hoc* analysis explained why these differences emerged; patients who benefitted from left-hemispheric inhibition had experienced more extensive lesions in the frontal cortices than patients who responded to the typical paradigm of left-hemispheric facilitation. Overall, remapping of hemispheric specialization of language subfunctions served as a better guide to identify an alternate approach for recovery in aphasia than the traditional approach based on a generic model of interhemispheric inhibition.

While the sample was small, we discuss Hamilton et al.'s model [138] here because it can be exemplary for individualizing stimulation for upper limb recovery. Still, provisions would have to include a triage process that involves crossover comparisons, where one would have to identify which application best suits each individual. Larger number of patients would be required so best responses are discerned across larger samples. Models such as this, however, could forego systematic triage if it were possible to predict* a priori* who would respond to which type of stimulation. Models discussed below offer such opportunities. Regardless, individualizing stimulation based on patients' own response to different types of stimulation is systematic and patient-driven.

### 5.2. Unimodal Models Predicting Recovery Based on Patient Characteristics

**Figure 3:**
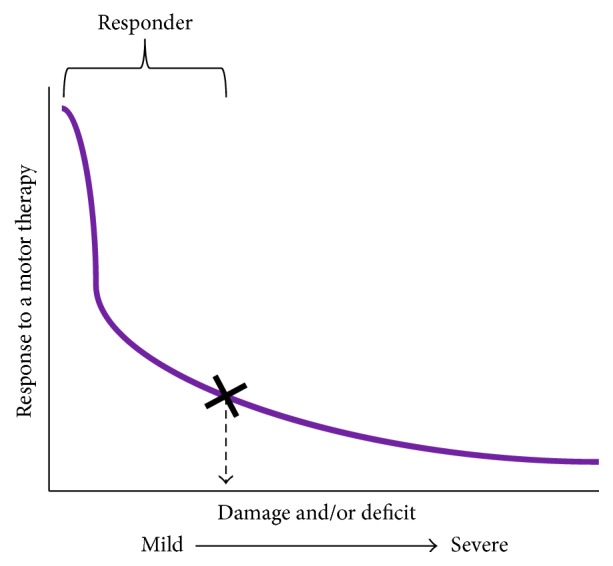
Presenting a schematic of unimodal models of recovery. Typically, unimodal models show how recovery following a motor therapy varies as a function of patient's individual characteristics, like damage to ipsilesional pathways, or impairment of the paretic limb. When characteristics are plotted against patient's response to motor therapy, one can understand who achieves criterion level of recovery (marked by X). Patients who achieve at least the criterion level or greater recovery are known as “responders.” Others are considered to have hit the “point of no return” (see Stinear et al. [81]). Degree of damage or deficit (or any other patient characteristic) that separates responders from nonresponders is deemed as cut-off to stratify patients for said therapy. It is important to note that criterion level of recovery, hence the cut-off, can vary from one therapy to another therapy and from study of one characteristic to another. If extrapolated, such recovery models can be effective at predicting who would respond to stimulation of ipsilesional M1.

Perhaps, the most common models are models predicting recovery. There has been a growing interest to predict who recovers and who does not recover after a stroke. One might further argue that these existing models can be extrapolated to explain who recovers from stimulation of ipsilesional M1 and who does not recover. Models prognosticating chronic recovery like those by Stinear et al. [81, 142, 143], Crafton et al. [144], O' Shea et al. [145], and Quinlan et al. [146] are based on a simple yet powerful* premise*. Knowing baseline characteristics that govern recovery can potentially help stratify patients for stimulation of ipsilesional M1. In separate studies, investigators examined patients who underwent motor therapies for the paretic upper limb [81, 142–144, 146] or traditional stimulation therapy [145]. Assessment of baseline characteristics included clinical scales of motor impairment, functional activation (fMRI) and functional connectivity, damage to corticospinal integrity studied with DTI, and excitability of descending pathways studied with TMS. Other variables included demographics (age, sex, and handedness), nature of stroke (lesion volume (cc); damage to motor cortices; location, i.e., cortical/subcortical/mixed; ischemic/hemorrhagic; side of stroke), neurologic status (chronicity, paresis of dominant side, cognitive function, depression, neglect, and aphasia), and comorbidities (hypertension, diabetes, hyperlipidemia, and smoking) [46, 56, 146, 147]. Bivariate and multivariate analyses explained which baseline characteristics predicted recovery with motor therapies [81, 142–144, 146] or with stimulation of ipsilesional M1 [145]. Overall, models showed that potential for recovery decreases with incrementally greater damage (Figure 3). Crafton et al. [144] recommended that patients showing >37% loss of fMRI activation in ipsilesional M1 experience greater motor impairment. Quinlan et al. [146] extended these findings, suggesting that patients experiencing >63% injury to corticospinal tracts (studied with DTI) cannot achieve significant gains with motor therapy. O'Shea et al. reported only patients with better baseline function and greater chronicity most respond to typical stimulation where contralesional cortices are suppressed (*R* ^2^ = 52.8%) [145]. In a separate study, Stinear et al. [81, 142, 143] used a similar multivariate model, but with a multilayered scheme. In patients who could evoke potentials in paretic muscles with TMS, they found excitability of descending pathways predicted recovery (*R* ^2^ = 58%), but, in patients who did not show any response to TMS, residual integrity of corticospinal pathways captured with DTI predicted recovery (*R* ^2^ = 67%). Patients with the worst levels of residual integrity however (worse than a cut-off of DTI value of 0.25) were considered to have hit a “point of no return”; that is, they showed very little prospect for gain with unilateral motor therapies (*R* ^2^ = 71%). Stinear et al. utilized a decision-tree to explain how such models could be extrapolated to predict who would respond to stimulation of ipsilesional M1. Overall, patients with spared ipsilesional M1 and pathways, that is, those below a “point of no return” (or those who have suffered <37% loss of activation of ipsilesional M1 or lost <63% of corticospinal tracts), are candidates for stimulation of ipsilesional M1.

Recovery-based models are powerful because by showing how recovery decreases exponentially at a set level of damage (or threshold or cut-off level of another characteristic), they can stratify patients for stimulation of ipsilesional M1. According to these models, patients below the cut-off or the threshold of injury recover best from motor therapies. Therefore, these models are unimodal because the peak (mode) of recovery lies below the threshold. As such, recovery-based models are superior to cross-sectional studies or studies requiring systematic comparison of differing stimulation therapies [139] because thresholds derived in a single study can help stratify candidates in future studies.

Important caveats need to be considered, however. We have, for instance, witnessed that patients with wide-ranging corticospinal injury respond to intensive motor therapies; intensive therapies likely have an equalizing effect across patients with mild as well as substantial corticospinal injury [67, 68]. Cut-offs or thresholds of injury derived in unimodal recovery models therefore may vary with the nature and intensity of therapy. Further, unimodal models are unable to directly test what alternate options exist for patients who suffer from greater-than-threshold level of injury. Finally, in multivariate models, it is important to derive weights for predictors, in this case, weights for the different characteristics. This would explain how to stratify patients based on not just one, but a combination of baseline characteristics, including corticospinal tract injury [81, 142, 143, 146], loss of fMRI activation [144], baseline function and chronicity [145], cortical/subcortical nature of stroke [56, 57], and excitability of contralesional cortices [57] to name a few.

### 5.3. Bimodal Model Predicting Recovery Based on Patient Characteristics

Bimodal models, such as one recently proposed in a landmark study by Di Pino et al., best complement unimodal recovery models [148]. Bimodal models differ from unimodal models because not only do they hypothesize how peak (mode) of recovery with a therapy will lie below a certain threshold of injury, but they also explain how patients above the threshold could benefit from an alternate therapy. The* essential premise* of the most recent bimodal model, called the bimodal balance-recovery hypothesis, is that the* structural reserve* is the most important patient characteristic dictating individual expressions of plasticity. If ipsilesional corticospinal pathways are structurally viable or spared, then patients could recruit ipsilesional M1 and its pathways and benefit from the standard stimulation of ipsilesional M1 and suppression of “inhibitory” contralesional M1. But, if the ipsilesional pathways are damaged substantially, then contralesional cortices would become adaptive rather than becoming inhibitive and could be stimulated to support recovery. Partial support for Di Pino et al.'s bimodal model comes from studies suppressing contralesional cortices. Patients experiencing lesser damage respond well to typical suppression of contralesional cortices, but patients with excessive damage instead experience deterioration of function, suggesting that contralesional cortices support their recovery against tenets of classical model of interhemispheric inhibition [93–95]. As such, the bimodal view helps clarify long-standing speculations about the variable role of contralesional cortices. A bimodal model also extends knowledge beyond recovery-based hypotheses explaining how traditional approaches may benefit patients with reasonable residual integrity but a new approach that involves facilitating contralesional cortices could theoretically benefit patients with greater damage. The latter possibility and as such the bimodal model here remain untested in humans, though a recent study shows promise of facilitating contralesional cortices in animal models with large infarcts [149].

### 5.4. Bimodal Model Based on Inherent Expressions of Plasticity

**Figure 4:**
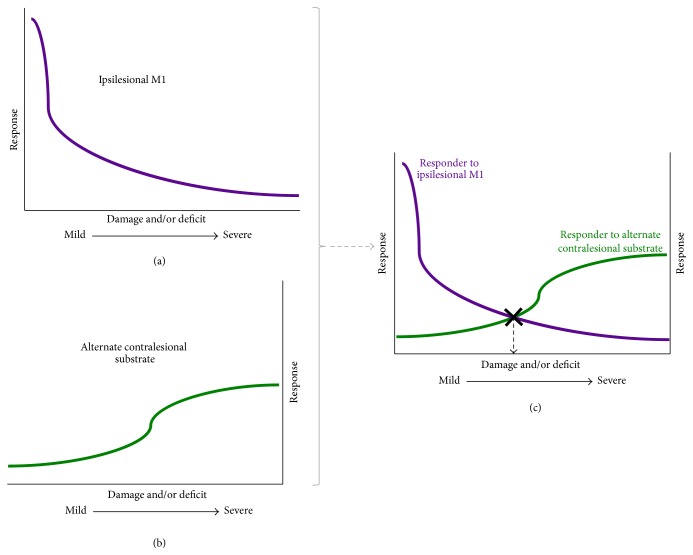
Bimodal model based on inherent expressions of plasticity. We propose a bimodal model that explains how to empirically derive a cut-off that separates responders for stimulation of the traditional substrate-ipsilesional M1 (a) versus stimulation of an alternate substrate in the contralesional cortices (b). Our proposed bimodal model of paretic upper limb recovery: cut-off derived empirically (c).

Here, we extend the hypothesis proposed by Di Pino et al. Specifically, we explain how to derive the cut-off or structural reserve or neural threshold of injury that differentiates between ipsilesional and contralesional expressions of plasticity. Our* premise* is that stimulation would be most effective if it boosts patient's mechanism of plasticity. To derive a robust model, we anticipate requiring a series of 2 studies, which are paired. The first study will adopt a crossover design, where patients receive stimulation of the standard target-ipsilesional M1 and stimulation of an alternate target in the contralesional cortices, besides sham. Stimulation will be offered for a single session each, where adequate time is allotted between sessions for washout of effects. The choice for alternate target in the contralesional cortices is described in detail in previous sections; for instance, cPMd would potentially be an ideal region to target based on evidence from other studies and the theoretical framework established in Figure 2 [94, 95]. Patients will be tested upon improvement of timed functional activities of the paretic upper limb, activities that are responsive to change within a single session in patients with mild as well as severe disability. Improvement with standard stimulation of ipsilesional M1 will be studied as a function of baseline damage and impairment. Improvement with stimulation of cPMd too will be studied as a function of baseline damage and impairment. If Di Pino et al.'s hypothesis is accurate, then we would anticipate improvements with standard stimulation of ipsilesional M1 will reduce with greater damage and impairment, whereas improvements with stimulation of cPMd will increase. Based on their opposing variances, we would be able to identify the intersection (Figure 4) that would serve as the cut-off level of damage and impairment that stratifies patients for tailored therapies.

The second study in our series will advance significantly beyond Di Pino et al.'s hypothesis to generate a more robust model to tailor brain stimulation therapies. Patients from the first study will participate in a second study after a period of washout. In the second study, they will receive rehabilitation for the paretic upper extremity. No stimulation will be provided. The goal will be to observe processes of plasticity elicited in recovery. We would observe pre- to-postchanges in excitability of ipsilesional M1 and ipsilesional pathways and changes in excitability of cPMd and changes in its inhibition on ipsilesional M1. We will study if plasticity of iM1 and ipsilesional pathways reduces with greater damage and impairment of the paretic limb. Similarly, we will study whether plasticity of cPMd potentiates with greater damage and impairment. Based on their opposing variances, we will be able to identify the intersection between plasticity of ipsilesional M1 and cPMd. This cut-off of plasticity, derived from the second study, will be compared to the cut-off derived from the first study. We will examine whether responders to stimulation of ipsilesional M1 in the first study express greater plasticity of ipsilesional M1 than plasticity of cPMd. We will also study whether responders to stimulation of cPMd express greater plasticity of cPMd than ipsilesional M1 in their recovery. Therefore, the second study will help us confirm whether plasticity witnessed in individual recovery over several sessions of rehabilitation validates cut-offs derived from single sessions of stimulation in the first study. As such, the second study will help confirm the model for tailored stimulation derived from the first study. More importantly, the second study will allow us to modify cut-offs derived from the first study. Therefore, our series will stratify candidates for standard stimulation of ipsilesional M1 versus novel stimulation of cPMd based on evidence of their plasticity observed in long-term recovery. Once the series is complete, then future studies can simply follow our stratification guide to test tailored stimulation. Thus, our series will not need to be repeated in subsequent studies.

Our model that stratifies patients based on a bimodal model of ipsilesional versus contralesional plasticity is conceptually different still from Di Pino et al.'s model because of the following.

(i) While we will validate Di Pino et al.'s hypothesis in the first study of our series, our series will be unique because it will empirically derive the cut-off or “structural reserve” to stratify patients for different therapies.
(ii) Compared to Di Pino et al.'s model, our model is validated by expressions of plasticity. We propose a model derived from paired studies, where we will confirm that patients who recover with stimulation of the standard ipsilesional M1 recover via plasticity of ipsilesional M1 and patients who recover with stimulation of cPMd recover via plasticity of cPMd.
(iii) A model that is based on both response to stimulation and long-term plasticity will likely be more robust to tailor stimulation therapies.

Because bimodel models, like Di Pino et al.'s model and our own model, compare two alternate therapies unlike unimodal models, they help validate the role of contralesional cortices in recovery. For instance, we typically suppress excitability of contralesional cortices assuming they compete with ipsilesional M1 [13, 24, 25]. But, it is likely that they support recovery in patients with greater damage [38, 80, 94, 95, 148]. Bimodal models are set up to clarify these theories. In patients ranging from mild to severe damage, if patients with greater severity benefit from stimulation of contralesional cortices but fail to benefit from their typical suppression, then it would confirm that contralesional cortices are supportive and not inhibitive in patients with greater severity.

According to the models discussed here, response to individual treatments can be predicted on the basis of measurable “parameters” or characteristics that differentiate between patients. These models collectively propose that defining heterogeneity in terms of characteristics allows one to understand who would potentially express which mechanism of plasticity in recovery. Such knowledge of individual mechanisms could guide personalization of stimulation. A key drawback however lies in the assumptions of plasticity. What if the treatment tested in unimodal models or two alternate treatments tested in bimodal models are not relevant to a patient's recovery? The model below developed in the context of depression could help address the caveats of existing models in upper limb recovery.

### 5.5. Models Based on Network-Based Connectivity

Even though recovery-based models and the bimodal hypotheses can empirically explain how to identify who would respond to stimulation of one region versus another, there is still a degree of uncertainty. Can patients be reasonably and clearly considered to fall into one or the other categories? Here is where a model recently employed in depression can be particularly informative. This* model considers* that neurological diseases like stroke, Parkinson's disease, and so forth can be conceptualized as diseases of networks rather than of unitary brain regions [150]. Interactions across networks can be witnessed using techniques like resting state functional connectivity MRI (rs-fMRI) that study polysynaptic connectivity across immediate and remote networks. The model has been studied more extensively in depression [151, 152]. The usual suggestion is to target the region of dorsolateral prefrontal cortex (DLPFC) commonly believed to be located 5 cm anterior to the site in M1. But, such an unvarying approach can evoke variability. This issue plagues the field of depression. A possible remedy is to study group-level hypometabolism in the region of DLPFC. But, targeting such regions has been ineffective as well. Based on previous studies suggesting that sites in DLPFC that are most effective are functionally connected with subgenual activity, Fox and Pascual-Leone et al. [150, 151, 153] have proposed an elaborate model to individualize stimulation to the DLPFC. Subgenual connectivity is used as a guide to target stimulation to DLPFC.

One can extrapolate this concept to the development of better strategies to improve upper limb recovery in stroke. It can be envisioned that regions showing highest connectivity to ipsilesional M1 would potentially be well positioned to support recovery. Since the investigation would be network-wide, it would decrease our reliance on one or another substrate of recovery and create opportunities for individualizing stimulation across many.

The key points to remember, however, are the potential limitations of the model if it is directly applied to stroke. Challenges presented in stroke are unique compared to depression and neurocognitive diseases [150, 151, 153]. For instance, relying on a perfusion-based contrast alone can be problematic in stroke since localization of activation is contorted in areas of vascular compromise [154, 155] and can shift inconsistently in recovery [154]. Most importantly, recovery-based unimodal models have taught us that structural integrity of corticospinal tracts is key for stroke recovery [81, 142, 146]; fMRI activation is generally epiphenomenal to their integrity [23, 156–158]. As such, one may have to be cautious in interpreting the exact location of rs-fMRI activity and may have to factor in residual integrity and baseline abilities.

## 6. Conclusions

A great challenge facing stroke rehabilitation is the lack of information on how to derive targeted therapies. As such, techniques once considered promising, such as brain stimulation, have demonstrated mixed efficacy across heterogeneous samples in clinical studies. Here, we explain reasons, citing its unvarying assumption and a one-type-suits-all approach as the primary cause of variable efficacy. We present evidence supporting the role of alternate substrates, which can be targeted instead in patients with greater damage and deficit. A significant roadblock, however, is the lack of information on how to tailor brain stimulation therapies and how to stratify patients for stimulation of traditional versus an alternate substrate for recovery. To this end, we discuss different frameworks. To the best of our knowledge, our report is the first instance that enumerates and compares across theoretical models from upper limb recovery and conditions like aphasia and depression. In agreement with Ward et al. [23], we explain how different models capture heterogeneity across patients and how they use heterogeneous patient characteristics to predict which patients would best respond to what treatments to develop targeted, individualized brain stimulation therapies. Our intent is to weigh pros and cons of testing each type of model so brain stimulation is successfully tailored to maximize upper limb recovery in stroke.
